# Challenges Inherent in Controlling Prickly Pear Species; a Global Review of the Properties of *Opuntia stricta, Opuntia ficus-indica* and *Opuntia monacantha*

**DOI:** 10.3390/plants11233334

**Published:** 2022-12-01

**Authors:** Talia Humphries, Shane Campbell, Singarayer Florentine

**Affiliations:** 1 The Future Regions Research Centre, School of Science, Psychology and Sport, Federation University Australia, Mount Helen, VIC 3350, Australia; 2 School of Agriculture and Food Sciences, The University of Queensland, Gatton, QLD 4343, Australia; 3 Applied Chemistry and Environmental Science School of Science, STEM College, RMIT University, 124 La Trobe St, Melbourne, VIC 3000, Australia

**Keywords:** invasive *Opuntia* species, *Opuntia stricta*, *Opuntia monacantha*, *Opuntia ficus-indica*, prickly pear management, weed managemnet, biological control

## Abstract

*Opuntia* species (prickly pear) were deliberately introduced to many countries around the world for fruit, cochineal dye production, living fencing or as ornamentals. They are now some of the world’s most significant weeds, particularly in regions with warm and or dry climates, as they pose threats to economic and environmental assets. In addition, they can cause considerable health issues for humans and animals. *Opuntia* spp. have prolific reproduction abilities, being able to reproduce both vegetatively and by seed. They have generalist pollination and dispersal requirements, which promotes their establishment and spread. *Opuntia stricta, O. monacantha* and *O. ficus-indica* are the most globally widespread of the *Opuntia* spp. In many countries, biological control agents, particularly the cactus moth (*Cactoblastis cactorum*) and various cochineal insects from the *Dactylopius* genus, have successfully reduced land-scape scale populations. On a smaller scale, controlling these weeds by either injecting or spraying the cladodes with herbicides can provide effective control. Care must be taken during herbicide treatments as any untreated areas will regenerate. While biological control is the most cost and time effective control method for landscape-scale infestations, further research into the combined efficacy of herbicides, fire, grubbing and pre-burial techniques would be beneficial for land managers to control small-scale and establishing populations. It would also be useful to have greater knowledge of the potential seedbank longevity and seed ecology of these species so that integrated management strategies can be developed to not only deal with initial populations but also the subsequent seedling regrowth.

## 1. Introduction

*Opuntia* species are the largest clade within the Cactacaeae family, and are native to both American continents. They have evolved many adaptive strategies that allow them to thrive under extreme climatic and environmental conditions, including extreme heat, low water availability and salinity [1]. This is mainly due to their exceptional water-conserving strategies, including the ability to photosynthesise with minimal transpiration by utilizing the Crassulacean Acid Metabolism (CAM) photosynthetic pathway [2]. In this process, the enzyme phosphoenolpyruvate (PEP) bonds a CO_2_ molecule to a 3-carbon sugar to produce the storable compounds; malate or oxaloacetate. These pH-reducing compounds are readily stored within vacuoles until the presence of sunlight. When the malate or oxaloacetate leave the vacuole to be used for photosynthesis, the compounds are decarboxylated and the carboxyl (CO_2_) is released at the rubisco activity site. The benefits of being able to store CO_2_ in this manner allows for the cells to open their stomata when conditions are cooler, usually during the night or at times with low vapour pressure deficit, such as dawn or dusk, which means that the stomata can remain closed during the heat of the day when the sun is most intense without sacrificing photosynthetic activity. This water-conserving strategy provides a competitive advantage in hot and/or water-limited environments throughout the world [3].

Three species belonging to the *Opuntia* genus; *Opuntia stricta* (Haw.) Haw., *Opuntia ficus-indica* (L.) Miller. and *Opuntia monacantha* Haw. (Cactaceae) are of particular concern at a global scale due to their aggressively competitive nature, and the high degree of difficulty in controlling them. Once established, they can cause a significant reduction in the carrying capacity of agriculturally important landscapes, and reduce biodiversity in areas with natural growth [3,4,5,6,7,8].

It is known that deliberate introductions of exotic species generally have significantly higher establishment rates than accidental introductions [9]. In this regard, in almost all situations, *O. stricta, O. ficus-indica* and *O. monacantha* were deliberately introduced for fruit cultivation [10,11], cochineal dye production [12], living fencing [13,14], fodder [15] or as ornamentals [16]. These domesticated cultivars frequently lack glochids and pose low risk to human or animal welfare, but escaped domesticated cultivars have been observed to revert back to their wild, prickly form [17].

Biological control programs targeting *Opuntia* spp. have been highly successful in many countries. Natural enemies to *Opuntia* spp., particularly the cochineal insect (*Dactylopius* spp. Costa.) and to a lesser degree, the cactoblastis moth (*Cactoblastis cactorum* (Berg)), have been introduced to assist in significant reductions of *Opuntia* spp. in densely invaded areas [3]. However, many of these biological control agents target just one species within the *Opuntia* family [18], and in addition to the many successful introductions of biological control agents, there have been many failed attempts. Indeed, in some countries such as Portugal, a suitable biological control agent has not yet been identified [19]. In these cases, cultural control methods and herbicide applications are the only management options available. While these methods may be effective for controlling localised populations, without the introduction of a biological control agent, managing-landscape scale populations of these *Opuntia* spp. is unlikely to be successful [20,21].

*Opuntia* spp. have successfully established and widely spread across the globe and are considered invasive to countries within Europe, Oceania, Africa and Asia [3]. They are not normally problematic within the native range of North and South America, as natural predators and climatic conditions maintain the population densities; however, some management intervention is required when they encroach into rangelands, woodlands and roadsides as populations can quickly grow and threaten agricultural, environmental and infrastructure assets [2,5]. *Opuntia stricta* has a large native range which includes the Caribbean, the southern states of the USA, Mexico and the northern countries of South America [5]; however, Weniger [22] suggests this species was introduced to the South American countries. *Opuntia monacantha* evolved in South America and is considered native to Brazil, Argentina, Paraguay and Uruguay [8]. *Opuntia ficus-indica* is native to only Mexico and was considered a plant of cultural and spiritual importance to the Aztecs [7,12].

The purpose of this review is to consolidate information regarding the ecology, global distribution and current management of *O. stricta, O. monacantha* and *O. ficus-indica*. To our knowledge, no review has yet been specifically conducted on these three most aggressive *Opuntia* spp., and a clear understanding of their similarities and differences in their ecology, together with information on the dispersal and management history, will be important for identifying what research is required for their increased control in the future.

## 2. Global Invasive Distribution and Impact

Globally, the success of *O. stricta*, *O. ficus-indica* and *O. monacantha* is directly attributed to three key factors: (i) human aided dispersal; (ii) release from natural enemies; and (iii) generalist ecological traits. It is known that plants that are purposefully introduced into a novel environment have a greater chance of establishment success [9]. In almost all cases, *Opuntia* spp. were introduced into their invasive range by humans for cochineal dye production, living fencing, or fruit cultivation [10,11,12,13,14,15,16]. In the invaded range, the *Opuntia* spp. were released from their often specialist, host-specific predators [3,15]. Furthermore, the spread of the *Opuntia* spp. was facilitated by a wide-variety of animals, including pollinators and frugivores [3,6,7,8].

### 2.1. Europe

It has been speculated that *O. ficus-indica* was first introduced to Europe by the Spanish at the end of the 15th Century or in the early 16th Century [12]. It was considered a highly prized plant due to the magnitude of benefits it offered, but particular interest was taken in establishing the cochineal dye industry [12]. It was cultivated for this purpose throughout Spain and the Canary Islands, with the latter being very successful, and is still currently in operation [12]. The fruit was also highly regarded, and in the 1950s, *O. ficus-indica* was the third most cultivated produce in Sicily, second only to grapes and olives [12]. In Greece, it was first cultivated by Venetians in Crete and the Aegean Islands in the late 1600s, where it grew well in dry, rocky soils [11]. It is considered an important crop in Greece [11], Turkey [23] and Italy, where annual fruit exports exceed 12,000 tons [12,24].

Despite this impressive level of economic importance, *Opuntia* spp. are regarded as significant weeds throughout Europe [25], particularly in the Mediterranean countries including Spain, Portugal, France, Italy Greece, Croatia and Turkey [26]. *Opuntia* spp. do not appear to have established in Northern European countries, most likely due to the cooler climate and higher rates of precipitation in these areas [27].

In Spain and Portugal [18,28], *O. stricta* is most often found in abandoned fields and natural landscapes, particularly those in close proximity to urbanised areas, which suggests that humans are facilitating its dispersal [26]. The cochineal insect, *D. opuntiae*, was unintentionally introduced to Spain where *O. ficus-indica* was considered invasive [29]. The favourable Mediterranean climate promoted establishment and the cochineal insect has provided significant control of this species [29]. Despite *O. monacantha* being recorded as an invasive weed in Spain, France [26], Italy and the Czech Republic [7], there is very limited information regarding the impact of *O. monacantha* on agricultural and environmental assets, or how it is currently managed.

The higher annual rainfall in the Mediterranean, compared to its native range, has been linked to improved rates of germination and seedling establishment, which has contributed to them becoming significant weeds in this environment [30]. *Opuntia* spp. have been observed to outcompete native plants in Portugal’s coastal regions, and their dense thicket growth form makes them difficult and expensive to manage [18]. Successful biological control agents have not yet been found for Portugal, and control relies predominantly on manual removal during the wet season, and by glyphosate injections prior to fruiting [18]. In Greece, *O. ficus-indica* has high genetic diversity, further complicating any management efforts [31]. Research into using spineless cultivars of *O. ficus-indica* for climate mitigation in the Czech Republic found that these cultivars reverted back to their spiny, invasive wild form in only a few generations of escaping cultivation, suggesting their release would have devastating implications on the ecosystem [32].

### 2.2. Africa

South Africa’s Biodiversity Act 10 of 2004 categorises all *Opuntia* spp., with the exception of the spineless cultivars, as Category 1 weeds. The spineless variety of *O. ficus-indica* was deliberately introduced to at least five African countries (South Africa, Eritrea, Ethiopia, Madagascar and Somalia) for crops, ornamental use, emergency fodder, cochineal production and bee forage [13,14]. It was purposefully introduced to the Karoo region of South Africa in 1656 and spread beyond the cultivated area. These escaped cultivars were observed to quickly revert back to their glochid forming wild type [33], and by 1942, *O. ficus-indica* covered over 900,000 ha of the Karoo region, making it one of the most widespread weeds in the country [16,34]. The purposeful introduction of the ‘ficus’ biotype of *D. opuntiae* reduced the spread of the *O. ficus-indica* population to less than 100,000 ha [35,36].

*Opuntia stricta* is considered one of the most aggressive *Opuntia* spp. in South Africa [37]. It was introduced as an ornamental plant to the Kruger National Park, South Africa, in 1953 [32], but by the late 1990s it had invaded over 30,000 ha. It was introduced to east Africa in the 1950s and populations have recently increased throughout Kenya. It is currently considered invasive in 14 African countries [6], and is particularly problematic in Kenya [34] and Namibia [36]; suitable biological control agents for controlling this species are under investigation. *Dactylopius opuntiae* was first released in Lakipia County, Kenya, in 2014. Significant improvements were observed after three years, although the population remained localised to the initial introduction site [32]. This same cochineal was deliberately introduced to South Africa in 1997, 10 years after *C. cactorum* was first introduced. Over a 22-year monitoring program, no reductions in the population density of *O. stricta* were observed during the time only *C. cactorum* was present, but the addition of *D. opuntiae* provided significant, cost-effective control within three years [36].

*Opuntia monacantha* has spread throughout 19 countries in Africa [7], and is particularly problematic in east Africa. It was first identified in South Africa in 1772, and it was widespread by the 1890s [38]. In some cases, this species occupied 100% of the standing vegetation canopy, creating hazardous thickets and facilitating the establishment of other invasive species, including rats [4].

*Opuntia monacantha* was the first cactus to be successfully controlled in Africa with the implementation of a biological control program in 1913 [38]. Various *Dactylopius* spp. were first introduced in South Africa [38], and then to Madagascar [4]. Attempts to introduce *C. cactorum* as a biological control agent were hindered by heightened predation of the egg sticks and larvae, as well as the limited resources to implement the program compared to those available in Australia [39,40]. In Madagascar, the spineless variety, *O. ficus-indica*, was cultivated in place of *O. monacantha* to overcome objections to the latter’s removal from the local communities and to create competition to reduce the re-emergence of the less favourable species [4].

### 2.3. Asia

In 1924, Sri Lanka introduced the biological control agent *D. opuntiae* to control *O. stricta* and other invasive *Opuntia* spp. [41]. While this insect has successfully become established, *O. stricta* is a serious concern in Sri Lanka’s arid [42] and coastal areas [43], and has rapidly spread throughout Bundala National Park [43]. Several *Opuntia* spp. have become invasive throughout India, with *O. stricta* being of particular concern [6]. Two insects, *D. coccus* and *D. ceylonicus,* were released in 1795 for controlling *O. monacantha*, which was at the time widespread [44,45]. These insects had a significant impact on reducing this species’ population density, but were ineffective against *O. stricta* [46,47]. After the success of the cochineal insect in Sri Lanka, *D. opuntiae* was introduced to India in 1925 and provided significant control of *O. stricta*, clearing over 40,000 ha of invaded land [48].

While *O. ficus-indica* was purposefully introduced to India in the seventh century by the British for cochineal dye production and as a fruit crop species, it failed to establish, and therefore, it is not listed as an invasive species. The failure of these plantations to establish is associated with pest insects and multiple flooding events [47]. This finding suggests that in addition to purposeful, human-aided introduction, release from herbivory and pests plays an important role in the invasive spread of these *Opuntia* spp.

To date, three *Opuntia* spp. have been deliberately introduced to China [49,50,51]. *Opuntia stricta* was introduced in 1702, *O. monacantha* in 1625 and *O. ficus-indica* in 1645. They are all now classed as Group III weeds, defined as being invasive species that only occupy a small area and have low harmful impacts on humans and the environment [52]. *Opuntia ficus-indica* and *O. stricta* are now invasive across five provinces in China, while *O. monacantha* is invasive in six [42,49]. These three *Opuntia* spp. occupy different environments throughout China. *Opuntia monacantha* is often found growing on slopes near coastal areas, *O. stricta* is also mostly found at sea level growing in rocky or sandy soils, while *O. ficus-indica* is more suited to hot and dry valleys [52]. In addition, *O. ficus-indica* is widely grown for its fruit throughout China, but it is currently unknown the extent to which these cultivations have reached [38]. As these *Opuntia* spp. have escaped cultivation, it appears the populations are contained and are not spreading. *Opuntia stricta* is currently widespread in Yemen, where it is having a significant impact on both environmental and human health, and there is no access to controlling the populations using biological control agents [53].

### 2.4. Oceania

At least 20 *Opuntia* spp. are naturalized in Australia, all of which, with the exception of *O. ficus-indica* and *O. dejecta*, are on Australia’s ‘Weeds of National Significance’ list [54]. *Opuntia stricta* is the most widely distributed *Opuntia* spp. in Australia, and this species has been observed in all states and territories [55]. *Opuntia ficus-indica* has also been recorded in all states and territories, with the exception of Tasmania [56], while *O. monacantha* has only been recorded in Queensland, New South Wales, South Australia, Western Australia and Victoria [57].

*Opuntia monacantha* was the first species introduced to Sydney in 1787 from Rio de Janeiro, in the hope of developing a cochineal dye industry [15]. It was also introduced as an affordable fodder for livestock, living fencing, fruit production and ornamental purposes [15]. *Opuntia monacantha* increased at a prolific rate throughout Queensland, increasing from 10,000,000 acres (approximately four million hectares) in 1900 to 60,000,000 acres (over 24 million hectares) by 1925 [58]. In climatically favourable years, *O. monacantha* could increase its population size by over 10 million hectares [57]. *Opuntia stricta* was introduced to Scone, NSW, for cultivation in 1939, from which it escaped and consequently prolifically spread across the border into Queensland [59]. By 1843, it was a significant weed throughout Queensland, covering an area of 240,000 km^2^ [58,59].

*Opuntia ficus-indica* was also deliberately introduced to Australia in the 1840s for cultivation of its high-quality fruits [15]. It escaped cultivation and has adapted to a wide variety of environments including coastal sites, woodlands, grasslands and scrublands, being most climatically suited to the southern half of the continent [54].

In 1912, investigations into finding a suitable biological control agent was commissioned by the Queensland Government, which resulted in the successful introduction of the cochineal insect, *D. ceylonicus*. This insect provided significant control of *O. monacantha*, and almost resulted in its complete eradication from Queensland [60]. Soon after, *D. opuntiae* was introduced to target both *O. stricta* and *O. ficus-indica*, and displayed similar levels of success [55,56]. *Cactoblastis cactorum* was introduced in 1926, and the high density released in Australia resulted in almost complete eradication [54]. The combination of these biological control agents, in addition to cultural and chemical methods, have provided ongoing control of all *Opuntia* spp. in Australia.

Three *Opuntia* spp. are naturalized in New Zealand, including *O. monacantha* and *O. ficus-indica* [61,62]. *Opuntia monacantha* was introduced from southern Brazil and Argentina as an ornamental, where it escaped and was naturalized by 1855 [58]. *Opuntia ficus-indica,* on the other hand, was not considered naturalized until 2000 [62,63]. *Opuntia monacantha* is listed as a significant weed in New Zealand, but information detailing its environmental and economic impact is limited [62,64]. These *Opuntia* spp. have become problematic to New Zealand’s coastal areas and beaches, as they grow well in sandy soils and are tolerant to moderate levels of salinity [62,64], although cladode growth is significantly reduced by moderate salinity [63].

Many of the Pacific Islands have introduced various *Opuntia* spp. for cultivation, with *O. monacantha* being the most widely established species. Fiji and Samoa have declared *O. monacantha* to be a noxious weed [65,66]. This species has also been recorded as invasive in the Fijian Islands, New Caledonia and the Philippines [7]. In these areas, information regarding the extent of the species’ invasiveness is not stated [65,66]. *Opuntia stricta* is also considered invasive in New Caledonia and the Solomon Islands [65,66].

## 3. Ecology

### 3.1. Climate Suitability

*Opuntia* spp. are well suited to areas with low precipitation and high temperatures, and they can tolerate moderate salinity. The ability of these plant species to be competitive in these harsher environments has assisted in their spread throughout arid, semi-arid, Mediterranean and coastal climates, particularly in degraded and over-grazed landscapes [2]. They prefer shallow, well-drained soils, and have been found growing well in rocky and sandy areas [2,6,8]. Their distribution has been observed to be restricted by latitude and altitude [27], as frost usually kills the growing apical meristems, although some frost tolerance has been observed in *O. stricta* and *O. monacantha* [6,8], which has assisted in these species invading temperate climate zones where winter frosts are common.

### 3.2. Physical Description

*Opuntia* spp. have several ecological and biological attributes that promote their establishment and spread when introduced to a novel environment, with these features providing a particular advantage in arid areas. An important growth trait that is unique to *Opuntia* spp. is the growth form of the cladodes, which are flattened succulent stems that grow in a direction that minimises their surface area exposed to the sun at the hottest part of the day, allowing the plants to avoid excessive heat stress [2]. Between *Opuntia* spp., cladodes differ in shape, size and colour, which influences their growth form and cladode-detachment, which is a vital mechanism for asexual reproduction [2]. Their fast-growing, shallow root system is one of these key adaptive strategies [2,67]. Within two years, their roots can spread 2.5 m from the plant’s stem, which allows *Opuntia* spp. to efficiently absorb water from light rainfall events [1,67]. Furthermore, during significant rainfall events, fine, temporary ‘rain-roots’ are rapidly produced to assist in additional water uptake [67]. Glochids are fine, hair-like spines that are present in wild *Opuntia* spp. and can provide some protection against herbivory. Due to the risk that glochids pose to the health of humans and animals, they are often absent or reduced in cultivated varieties [24,25,26,27,28,29,30,31,32,33,34,35,36,37,38,39,40,41,42,43,44,45,46,47,48,49,50,51,52,53,54,55,56,57,58,59,60,61,62,63,64,65,66,67,68]. The glochids play an additional role in harvesting water by collecting and channelling it towards the plant [69]. *Opuntia* spp. have also developed strategies that significantly reduce water loss from transpiration. These include (i) producing spines in place of leaves to reduce surface area, (ii) exuding a waxy coating to protect the cuticles, (iii) inducing a metabolic shutdown process called aestivation during summer to conserve energy, and (iv) using a CAM photosynthetic pathway, which allows for photosynthesis to occur with minimal transpiration [2].

### 3.3. Physical Description of Opuntia stricta

**Figure 1:**
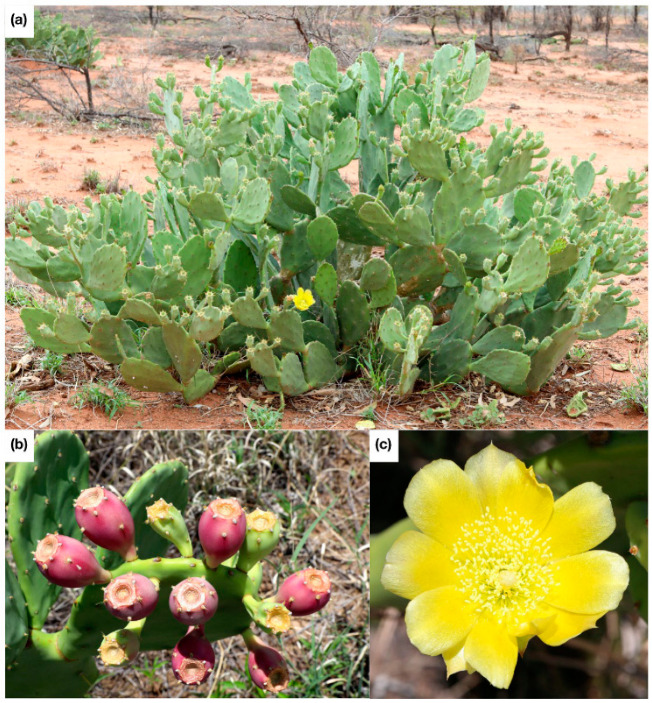
Photographs of *Opuntia stricta*. (**a**) depicts an image of the cladodes and growth form; (**b**) shows a close up of the fruit; and (**c**) is an image of the flower. Photographs provided by the Queensland Government (personal communication, 5 October 2022).

The dull, grey-green cladodes of *O. stricta* are 10–25 cm long and have an elliptical to obovate shape, and rather than growing tall (the plant only reaches 2 m), it has a sprawling shrub-like growth form, allowing it to rapidly spread across the landscape (Figure 1) [6,54]. This lateral growth form contributes to the hostile invasiveness of this weed, as once it is established, it smothers light and space for competing plants more rapidly than those with vertical growth. This species is considered the most aggressive *Opuntia* spp. worldwide and is listed among “100 of the World’s Worst Invasive Alien Species” [70]. *Opuntia stricta* has approximately 80 glochids surrounding each areole, which are 5 mm in length [69]. *Opuntia* spp. produce one sessile flower at the areole, and these are often yellow to orange for *O. stricta* [54].

### 3.4. Physical Description of Opuntia monacantha

**Figure 2:**
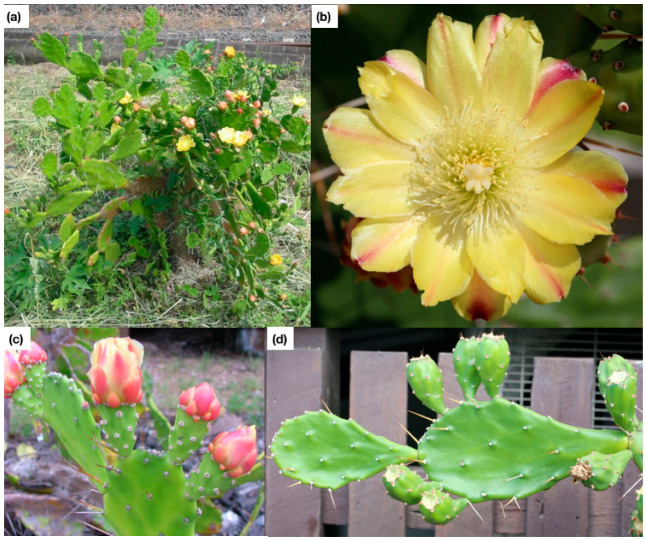
Photographs of *Opuntia monacantha*. (**a**) shows the growth form of a single plant; (**b**) shows a close up of the flowers; (**c**) shows a close up of a cladode with fruit and spines; and (**d**) highlights the narrow cladode attachment point. Photographs provided by the Queensland Government (photos b and d, personal communication, 5 October 2022) and Sheldon Navie (photos a and c, personal communication, 11 October 2022).

*Opuntia monacantha* has larger cladodes than *O. stricta*, ranging between 20–30 cm in length. This species is colloquially known as drooping prickly pear, and as the name suggests, it has a drooping appearance [15]. Despite this, the bright green cladodes are strongly attached, allowing it to grow up to 3.5 m in height (Figure 2) [8,54]. *Opuntia monacantha* produces mostly spineless, green to red fruit from yellow flowers [8,54].

### 3.5. Physical Description of Opuntia ficus-indica

**Figure 3:**
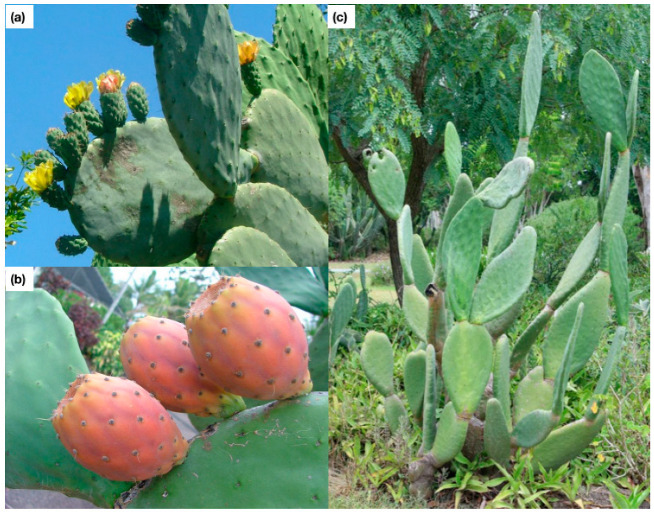
Photographs of the cladodes with emerging flowers (**a**), and fruit (**b**), as well as the growth form of *Opuntia ficus-indica* (**c**). Photographs provided by Queensland Government (photo c, personal communication, 5 October 2022) and Sheldon Navie (photos a and b, personal communication, 11 October 2022).

*Opuntia ficus-indica* has similar shaped cladodes to *O. stricta*. They are a pale blue-green colour and are considerably larger (20–60 cm long) and more firmly attached, giving it a tree-like structure and allowing it to reach up to 5 m in height (Figure 3) [7,54]. The large cladodes of *O. ficus-indica* have high concentrations of mucilage, which is a water-absorbing carbohydrate that allows the cladode to hold higher volumes of water [71]. This contributes to its success in ecosystems prone to drought [71]. While this species is globally cultivated and these domestic varieties are often free of glochids, wild *O. ficus-indica* have small (10 mm long) glochids which return within several generations of escaping domestication [32]. The flowers of *O. ficus-indica* are bright yellow, red or orange and can reach 9 cm in diameter [54,72,73]. The colour of their fruit can also vary, showing similar colours to the flowers, and are mostly spineless or have spines that are very fine and easy to remove. Fruit maturation occurs in late summer for *O. ficus-indica*, approximately 30–70 days after anthesis, and each fruit contains upwards of 200 viable seeds [74].

## 4. Sexual Reproduction

### 4.1. Pollination

Floral buds begin to emerge from areolas during the spring months for *O. ficus-indica*, which are triggered as a result of increased average daily temperature (at least 14 °C) and extended day lengths (of at least 12 h) [34]. Anthesis occurs mid-spring to summer for all three species, and multiple pollination methods are viable, with allogamy being the most common [12,29,33]. *Opuntia* spp. are able to attract a diversity of pollinators to enhance allogamy, and *O. ficus-indica* flowers are visited by more than 50 insect species, with Hymenoptera, particularly bees, providing the highest pollination success [29,33,34,35,36].

The flowers are considered botanically perfect, meaning they contain both male (stamen) and female (carpel) reproductive organs [2]. It has been observed that the preferred sexual reproductive strategy is xenogamy [72]; however, some flowers are self-compatible, allowing for self-fertilization [2]. The ability to self-fertilize gives these *Opuntia* spp. an adaptive advantage when introduced to a new area, or areas where suitable pollinators are rare or absent. The benefits of producing viable fruit and seeds in a novel environment enhance their ability to attract animals to facilitate their spread [75].

### 4.2. Seed Dispersal

*Opuntia* spp. produce brightly coloured fruits that attract a diversity of birds, reptiles and mammals for wide-scale seed dispersal [33,34,37,40,76]. Throughout Africa, a wide variety of animals have contributed to the rapid expansion of *Opuntia* spp. It was observed that elephants can transport seeds up to 15 km ahead of their current invasive range [34], and many frugivorous birds dispersed seeds over great distances, allowing these species to reach islands [76]. The presence of arils on *Opuntia* seeds also attracts dispersal and burial by ants (myrmecochory), which may act to protect the seeds from opportunistic predation [77].

### 4.3. Seed Dormancy

The thick seed coats in *Opuntia* spp. provide a mechanical barrier that prevents the protrusion of the radical, resulting in physical dormancy [78]. Over 90% of *O. stricta* seeds demonstrate long-term persistence, with the seeds remaining viable through dormancy for up to 20 years [16,74], and *O. monacantha* seeds have been observed to remain viable for up to 15 years [72].

### 4.4. Seed Germination

*Opuntia* spp. have been observed to have low rates of seed germination under both laboratory [77,78,79] and natural field conditions [80]. Additionally, rates of seed germination for *O. ficus-indica* [78] and *O. stricta* [18] were enhanced by light, but Podda et al. [30] found no significant difference between alternating photoperiods and complete darkness for *O. ficus-indica*. Increasing concentrations of salinity has been shown to reduce seed germination of *O. ficus-indica* but, nevertheless, the seeds were able to tolerate moderate salt concentrations and did not lose viability under high saline concentrations, indicating they could germinate and flourish in coastal regions [30]. While *O. ficus-indica* has observed low germination rates in the wild, domesticated varieties with high viability and vigour have been selected, demonstrating accelerated imbibition rates [81].

Alternating temperature regimes of 30/20 °C, scarification and sufficient water availability have provided the highest germination rate (approximately 80%) in *O. stricta*, which would coincide with spring and early summer conditions [16]. Often, scarification of the seeds can be achieved through the process of the seeds passing through the digestive tract of animals [76]. This was, however, not the case for *O. stricta*, which had significantly reduced viability after consumption by animals [76], suggesting other environmental disturbances, such as fire, may provide important germination cues for this species. It has been found that scarification of *O*. *ficus-indica* [30,78], *O. stricta* [16] and *O. monacantha* [72] seeds under laboratory conditions improved germination rates.

### 4.5. Vegetative Reproduction

To increase reproductive odds in an arid environment, *Opuntia* spp. are able to vegetatively reproduce, whereby any part of the parent plant with areoles that break away will develop roots and grow into a new plant [74,82]. Whilst this most often involves a cladode, vegetative reproduction from underdeveloped fruits has also been observed [2,16]. When areolas of the detached cladode come into contact with the soil surface, they will form roots and subsequently grow into a new plant [29].

Most often, cladodes will fall near the parent plant, attributing to dense populations, but it is possible for cladodes to be effectively dispersed via geochory and hydrochory. Spines can enhance dispersal by attaching to animals, boots and vehicles. Cladodes can survive for a considerably long period without sending out roots, and have remained viable for up to three years in a sealed container [72]. It is particularly relevant that vegetative reproduction has very high success rates in *Opuntia* spp., often with a 100% survival rate [74].

## 5. Management

The most appropriate control methods are often dependent on the invasion stage of the cactus infestation. Due to the prolific vegetative reproduction ability of these *Opuntia* spp., coupled with animal-assisted seed dispersal, the integration of multiple control methods is often needed to achieve sufficient control. This can include various combinations of cultural, chemical, manual and biological methods to kill standing plants, reduce the seedbank and prevent regeneration. Control efforts often take several years of consistent application before a significant reduction in the population can be observed, and thus, preventative measures should be ongoing.

### 5.1. Management Intervention for Early Invasive Stages: Introduction and Colonisation

The most ideal solution for targeting *Opuntia* spp. is to prevent invasion by maintaining a highly functional and competitive system. This was observed by Strum et al. [34], whereby *O. stricta* was not a problem weed for over 50 years in the Kruger National Park, South Africa, until the ecosystem underwent a state change caused by overgrazing and increased urbanisation, allowing the weed to establish and become widely spread. In addition to restoration and maintaining healthy land, invasive species hygiene practices are essential to prevent unintentional introductions. Hygiene practices, in this sense, refer to the measures taken to prevent the spread of invasive plant propagules by removing seeds and contaminated soil, water and organic materials from clothing, footwear, vehicles and equipment [83]. This includes inspecting vehicles and machinery after being used in an invaded site, using hay which is free of *Opuntia* seeds, and regularly inspecting the property and removing and immediately destroying any emerging *Opuntia* spp. [72,84].

### 5.2. Management Intervention for Intermediate Invasive Stages: Establishment

#### 5.2.1. Grazing

Livestock and wildlife can be used to reduce small populations of *Opuntia* spp. by grazing the cladodes [37,84]. In times of drought, consumption of cladodes can provide hydration as well as sustenance when other forage materials may be limited [84,85]. However, the spines and glochids can result in ulcerations and sores around the eyes and mouths of livestock, and providing supplementary feed will be required to prevent a build-up of glochids in grazing animals’ stomachs. In some cases, to encourage livestock to graze the *Opuntia* spp., land managers have used blow torches to singe away glochids, allowing livestock to graze with reduced physical harm [84]. It is unlikely that grazing alone would provide complete control of a moderate invasion, and it would be critical for any dropped cladodes to be sprayed to prevent vegetative reproduction, as well as monitoring the site for any seed germination that may have passed through the grazing livestock.

#### 5.2.2. Chemical Control

Arsenic pentoxide was first trialed for the control of *O. stricta* and *O. monacantha* in 1916 [5,6,27]. This herbicide has been replaced with more widely available herbicides, including glyphosate [19], MSMA [51], picloram and triclopyr, with the latter two often used in combination [54]. Herbicides can be sprayed directly on the foliage, but they must be applied with care as any areas missed will regenerate [3,54]. Furthermore, the uptake of herbicides is restricted by their thick epidermal cells, which prevent absorption of the herbicide, and this is further enhanced by the stomata remaining closed during the day, which is often the time of the herbicide application, reducing the rate of herbicide absorption [86]. To overcome these barriers, herbicides can be directly injected into either the topmost cladodes [19] or to the plant’s stem [87]. As *Opuntia* spp. often grow in unproductive locations that are unsuitable for cultivation, the cost:benefit ratio for control is often financially unviable, which results in many populations being left untreated [58].

#### 5.2.3. Grubbing

Manually removing *Opuntia* spp. is highly effective; however, doing so can pose a hazard to human health and safety. It is also important to consider the reproductive methods of *Opuntia* spp., as in most cases, they can either directly recolonise from the seedbank or vegetatively regenerate unless every part of the plant is removed [19]. As a consequence, the removed fragments must be treated and sufficiently buried to prevent regeneration [88]. Pre-burial treatments include soaking the plant fragments in water for 20 days to accelerate the rotting process, or burning the plant fragments [86].

#### 5.2.4. Fire

While cacti are not fire tolerant, their high-water content acts to resist fire and makes them difficult to ignite [89]. Therefore, in order for fire treatments to be most successful, they should be implemented when the plants are dry. Implementing fires when the plant is drier after the summer resulted in 96% mortality of *Opuntia* spp., compared to only about 50% mortality during a winter burn [20]. In the USA, aerial spraying of large infestations with Picloram (2–4 pints per acre) has been observed to dry the plants, and thus, promote more effective burning during summer [84].

### 5.3. Management Intervention for Late Invasive Stages: Landscape-Scale Spread

Within their native range, *Opuntia* spp. do not often become invasive due to natural enemies, such as grazing mammals, insects and diseases, that maintain population levels. The establishment of biological control agents to tackle landscape-scale infestations is the most economical solution for long-term control [90]. When infestations of *Opuntia* spp. are widely spread, grazing mammals are not suitable for control as they can assist in dispersal of seeds and vegetative segments, whilst dense populations can restrict movement and cause the animals injury. Several insects, fungi and diseases have been trialed for controlling various *Opuntia* spp. on a landscape scale. Amongst these, the cochineal insect (*Dactylopius* spp.) and the cactus moth (*Cactoblastis cactorum*) have provided the most significant control and have been the most globally adopted.

#### 5.3.1. Cochineal Insect

In many cases, the cochineal insect, *Dactylopius coccus* (from order Hemiptera and the Dactylopiidae family)*,* was globally introduced alongside *O. ficus indica* and other domesticated varieties for coccidoculture (cochineal breeding) to produce carminic acid, an important and diversely useful red pigment obtained by crushing the sessile females [91]. There are 11 known *Dactylopius* spp. in this monophyletic genus, all of which are parasitic of cactus plants [92]. These insects are native to North or South America and, like the *Opuntia* spp., have evolved to tolerate arid and water-limited environments [91]. Cochineal insects are attractive biological control agents as they display host specificity and feed on only one or a few closely-related *Opuntia* spp. [3,54,93].

**Figure 4:**
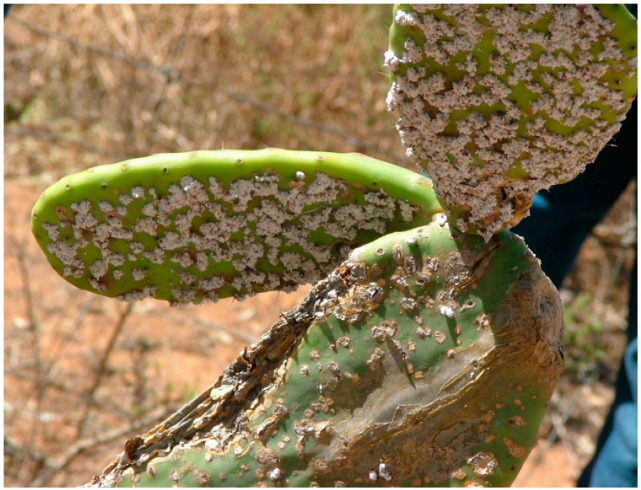
A photograph of the cochineal insect feeding on *O. ficus-indica* cladodes. The white, waxy coating produced by Dactylopius spp. is evident. Photograph provided by Sheldon Navie (personal communication, 11 October 2022).

Cochineal insects display sexual dimorphism and drastic differences are observed in the appearance and behaviour of adults [18,94]. The female cochineal insects have three life stages after hatching: the nymph stage, an intermediate nymph stage and the adult stage [18,95]. During the nymph stage, emphasis on finding a suitable feeding position is priority, and their bodies are covered in fine bristle to assist in wind dispersal, moving from a crowded cactus to a less populated plant [96]. During the intermediate stage, the insect inserts its mouth piece into the cactus and remains sessile in the spot for its entire life [18,60]. In this position, it then undergoes two malting events, where it develops a waxy coat for protection from harsh environmental conditions and predation [94,97], as shown in Figure 4. It enters the adult life stage after the second malting phase, where it reaches sexual maturity [94,95]. Male cochineal insects also undergo a similar nymph and intermediate nymph life stage; they develop wings in their adult life stage and are rarely observed feeding on the cacti [95].

*Dactylopius coccus*, as well as *D. austrinus, D. ceylonicus*, *D. confuses* and *D. opuntiae*, have all been trialled for the control of *O. stricta*, with only the latter providing extensive, widescale control, which has been subsequently exploited in Australia, Africa and the Middle East [36,98]. The ‘ficus-indica’ biotype of this species has also been identified to effectively target and control *O. ficus-indca* and provide reasonable, but not complete, control for *O. monacantha* [99]. Higher control levels were observed with *D. ceylonicus* for controlling *O. monacantha* throughout Africa and Australia [45,100].

The widely commercialised *D. coccus* has one of the broadest host ranges, and has been reported to feed on at least 14 *Opuntia* spp. [18,101]. This is the only cochineal insect that produces a high enough concentration of carminic acid to be considered economically viable for coccidoculture [18]. While this species feeds on a variety of invasive *Opuntia* spp., it is not a suitable biological control as it does not cause significant harm to adult plants [18].

Not all environments are suitable for establishing the cochineal insect as a biological control agent, and establishment has failed in some regions and countries [20,47]. Rainfall has a significant effect on colony survival of *D. opuntiae*, with 15 min of rain being enough to kill an establishing colony, and small colonies can be washed away after 120 min. In larger populations, at least 40% of the initial population was removed from the cladodes after 30 min of rain. Therefore, biological control is not suitable in areas that receive regular rainfall [102].

#### 5.3.2. Cactoblastis Moth

The phycitid moth, *Cactoblastis cactorum* (from the Lepidoptera order and Pyralidae family), is known for its successful control of *O. stricta* in Australia [100]. The *Cactoblastis* genus is native to southern South American countries, and *C. cactorum* is specifically native to Argentina, Uruguay and Paraguay. Unlike cochineal insects, the moth has a broader host range among *Opuntia* spp. [103,104]. Despite being native to South America, it has provided partial or complete control of invasive North American *Opuntia* spp., including *O. stricta* and *O. ficus-indica* [38].

The moths lay their eggs in a stick-like cluster on a cladode that is camouflaged to look like a cactus spine [105,106,107]. The emergence of the orange larvae occurs in unison, and this allows the insects to work together to break through the thick outer layer of the cactus and feed inside the cladodes and stems [38]. While feeding, *C*. *cactorum* work as a colony to tunnel and consume an entire cladode, leaving only the fibrous vascular layers untouched [18]. Mature *Opuntia* plants with woody cladodes are often less affected by the moths than younger plants with fresh cladodes, as the larvae struggle to break through the hardened outer layers [38].

In addition to the damage caused by larval feeding, the openings allow for diseases to enter the cactus and assist in killing the plant [108]. The cladodes detach from the plant, and these rotting cladodes provide the moth with shelter after they drop to the ground to pupate [18]. The emerging adult moths are inconspicuous, with brown to white wings and bodies [105,106]. The moths only live for nine days as adults due to their underdeveloped mouthparts, preventing them from feeding [38]. Therefore, dispersal is limited by this short life span of the adult females, and in Australia, *C. cactorum* populations have only travelled about 24 km from their site of establishment within 2.5 years, whilst in South Africa, they have only travelled 6 km within the same time frame [38,60].

In addition to suitable climatic conditions for the establishment of *C. cactorum*, population density is the biggest influence on the success of this moth as a biological control agent [109]. In Australia, *C. cactorum* provided excellent control of *O. stricta* due to the extensive resources available in terms of rearing facilities and volunteers, and approximately two billion egg sticks were released within three years [58,60,109]. It has been found that *Opuntia* spp. will survive low densities of the moths, where, for example, in South Africa’s Kruger National Park, the cladodes of *O. stricta* were only partially consumed, and when they detached from the parent plant, they were able to vegetatively regenerate, resulting in the population increasing [39].

The *O. stricta* plants in South Africa were also identified as being larger than the Australian population, which usually have less than 14 cladodes, and it is known that the moths are less affective against larger plants where the older cladodes are usually hardened, preventing the larvae from burrowing inside [110,111,112]. In South Africa, the cactus moth was also introduced to target *O. ficus-indica*, but it did not successfully kill the plants due to low density numbers [110]. It was found that the moths’ eggs and larvae were suffering higher predation rates from wildlife compared to those recorded in Australia, which prevented the population from reaching the required density [39,111]. However, the moth did cause enough damage to reduce the reproductive output of *O. ficus-indica*, and slowed the rate of the weeds’ spread [113]. Additionally, a small release of 60 egg sticks on *O. stricta* in the Kruger National Park had a noticeable impact on the population density, but did not provide complete control [109].

## 6. Key Findings

The global invasive spread of *O. stricta*, *O. monacantha* and *O. ficus-indica* has been facilitated by their purposeful introduction for crop cultivation and other human uses, as well as several key ecological traits that promote their dominance [114]. These key ecological traits and the associated management challenges are outlined in Table 1.

The key to the successful establishment of these *Opuntia* spp. is directly linked to the purposeful, human-aided introduction to almost every country where they are considered invasive. It is known that colonization pressure is increased for species that are purposefully introduced as they often have increased genetic diversity, with high numbers of individual plants or seeds being introduced to promote their establishment [115,116]. These *Opuntia* species were able to escape their cultivated range and establish wild populations via the effective dispersal techniques observed for these species, including: zoochory, hydrochory and attachment to vehicles, clothing and equipment. As a result of this, multiple populations were able to establish across landscapes free from natural predators and, as a result of the sharp spines and glochids, grazing animals avoided these species.

The glochids and spines also present significant health risks to humans and the dense thickets can prevent access to implement control actions, such as herbicide application, by foot. Aerial spraying *Opuntia* spp. populations with herbicide is often thwarted as a result of the thick waxy coat that reduces the herbicide uptake, and this is further restricted by the CAM photosynthesis that allows the stomata to remain closed during hot and dry weather conditions. It has been observed that invasive species that greatly differ from native species can have a competitive advantage [117], and CAM photosynthesis is unique to the Cactacaeae family and has significant advantages over the C_3_ and C_4_ photosynthetic pathways in water-limited and hot climates.

As a result of the release from natural enemies in their invasive range [118], coupled with the low grazing pressure from native mammals and livestock due to the hazardous glochid [119], these Opuntia spp. were likely able to increase their competitive ability through designating more resources to higher fecundity and growth [120,121].

Therefore, the introduction of biological control agents is the best solution for landscape-scale control of *Opuntia* spp. However, there are several challenges associated with establishing the two currently used genus of species. *Cactoblastis cactorum* has been successful only when high volumes of egg sticks are simultaneously released to target a population, while moderate volumes can only reduce the further spread. The challenges associated with the highly successful *Dactylopius* spp. include the sensitivity of these insects to cool climates and rainfall.

The final challenge associated with managing *Opuntia* spp. is associated with the long-lived, persistent seedbank, with seeds being observed to remain viable for up to 20 years. Due to the wide dispersal potential of the seeds, new populations could emerge in seemingly unaffected areas, or in areas that have previously successfully controlled the *Opuntia* spp. invasion. Any populations left unchecked to re-emerge from the seedbank would likely successfully establish as a result of the diversity of reproductive strategies these species can adopt, including sexual reproduction by xenogamy or self-fertilized flowerers, as well as vegetative reproduction. It has been noted that these *Opuntia* spp. seedlings are not competitive and they often invade after continuous ecosystem disturbance. However, re-emerging *Opuntia* spp. have been observed in areas of high, competitive, native grass cover [122], which adds further complexity to post-treatment restoration efforts.

## 7. Recommendations for Future Research

Much of the literature for controlling *Opuntia* spp. has focused on the discovery of suitable biological control agents, which has been seen to be successful in many parts of the world, and these efforts are enhanced when multiple species are introduced and cultural and chemical controls are combined [53]. In regards to the seed ecology for *O. ficus-indica*, *O. stricta* or *O. monacantha*, more research is required. Whilst light and temperature requirements have been studied, as well as factors affecting dormancy, investigation into other factors such as salinity, water availability, intermitted water availability, heat exposure, burial depth and seed longevity could assist in planning follow up management or understanding how to uniformly break dormancy or devitalise the seeds. Further research into fast and effective treatments to use on manually removed plants prior to burial would significantly enhance physical removal treatments, and the provision of more information on landscape-scale restoration projects that target sites invaded by *Opuntia* spp. would be beneficial for modelling similar future restoration projects.

## 8. Conclusions and Management Implications

The combined factors of prolific fruiting, strong vegetative reproduction and hazardous physical traits, with dense growth forms, spines and glochids, make *O. stricta*, *O. ficus-indica* and *O. monacantha* globally significant weeds. The findings of this review demonstrate that biological control agents provide the most effective landscape-scale control of invasive *Opuntia* spp., especially in degraded, nonarable areas, with dense populations quickly decimated when effectively applied. As *Dactylopius* spp. are species specific, they pose little threat to cultivated varieties or native vegetation. Due to the prolific vegetative reproduction of these species, after the implementation of biological control agents, it would be advised that any surviving cladodes be removed or treated with herbicide to prevent reestablishment. Further seedling emergence should be monitored for up to 20 years due to the persistent seedbank as a result of the seeds having mechanical dormancy due to the thick seed coats. In areas where biological control agents are not suitable, due to either cooler climates, high rainfall zones or limited funding, fire has been observed to successfully remove dense populations when implemented after summer, when these weeds are at their driest. It would be recommended to also treat cladodes that were not completely killed by the fire. Monitoring seedbank emergence would be critical as fire treatment may increase germination rate and uniformity, as fire is known to scarify hard-coated seeds. Once these *Opuntia* spp. are treated and removed, it would be beneficial to establish competitive and diverse native plant cover to outcompete seedlings for long-term control.

It is recommended that further research investigates the longevity of the seedbank of these three *Opuntia* spp. and ways to promote uniform germination to flush out the seedbank and target the emerging plants while they are in the juvenile form, as small plants are easier to kill than adults. Biological control should be further investigated for non-American countries that have not yet implemented successful introduction programs. Community awareness initiatives have been effective in reducing infestations of *Opuntia* spp. (as seen in Portugal and South Africa), and a focus to develop similar programs in other countries could also be highly beneficial.

## Figures and Tables

**Table 1:** Summary of key ecological traits of the three Opuntia spp. explored in this review, and how these traits facilitate their establishment, invasive spread and dominance in a landscape. The management challenges associated with these ecological traits are described.

Ecological Trait	Facilitates Invasion	Management Challenges
Generalist growth conditions	Tolerant to a broad range of climates and environments and can tolerate extreme heat as well as light frost.	*Opuntia* spp. populations can establish in different landscapes and environments, all which may require different management strategies.
	Bright flowers and fruit attract a variety of pollinators and dispersal agents.	
Glochids and spines	Protects cladodes from animals grazing, and can result in overgrazing of desirable plant species.	Glochids and spine cause hazards to human health and can limit access to control dense populations of *Opuntia* spp. Further, the spines can assist in cladode attachment to shoes, clothing, vehicles and equipment for dispersal, therefore hygiene practices are essential to prevent spread.
	Assists to harvest moisture in times of water scarcity.	
	Assists in dispersal of cladodes for vegetative reproduction.	
Reproduction	Utilizes both sexual and vegetative reproduction.	The ability for these weeds to vegetate poses a challenge to control actions, as all cladodes must be either removed, buried, or treated to prevent the plant re-establishing. The long flowering and fruiting season can make it and ongoing challenge to reduce seed production and dispersal.
	Cladodes can survive for an extended period on the ground before establishing roots.	
	High cross-pollination to improve genetic diversity.	
	Flowering and fruit production can continue for several months.	
	Some flowers are self-pollinating to ensure seed and fruit production in areas with small populations and/or limited pollinators.	
Low fire tolerance	The high-water content of *Opuntia* sp. makes them difficult to ignite. In areas with high population densities, fire regimes are reduced in frequency or intensity, and this reduces biodiversity and native regeneration from the seedbank.	Fire is often an effective restoration tool for fire-prone ecosystems. Fire is often an economical control action to implemented at a land-scape scale, however *Opuntia* spp. do not effectively carry fire and this treatment is only beneficial when the plants are dry, usually after summer.
CAM photosynthetic pathway	The stomata are often closed during the day, particularly on hot days to conserve water.	The stomata being closed throughout the day can limit the ability for herbicide to enter the plant.
	Reduced transpiration and photorespiration.	
Thick, waxy coating	The thick, waxy coating on the cladodes act to protect the cuticle and conserve water.	The thick, waxy coat of the cladode act to block herbicide application, which significantly reduces the effect of spraying herbicide. The alternative for land managers is to inject the plants with herbicide, however this is extremely time consuming and poses a significant health risk.
Fast growing and responsive root system	*Opuntia* spp. quickly establish fibrous root systems that can outcompete other plants for water. Temporary rain-roots allow for rapid uptake of water in areas of unpredictable rainfall.	Fibrous root systems make it challenging to remove plants through manual removal control actions such as grubbing.
Seed dormancy	The hard-coated seeds are able to persist in the seedbank for an estimated 20 years.	Long-lived persistent seedbanks pose significant ongoing challenges to treated landscapes. Seedbank recruitment needs to be monitored and any emerging seedling should be controlled for at least 20 years post control actions.
	The seed coat can be scarified by the digestive tract of animals. This promotes germination after dispersal.	
	Scarification can be also achieved by disturbance events, allowing seeds to germinate under low competition.	
Limited enemies outside native range	There are no natural predators to *Opuntia* spp. outside of its native range, which allows populations to grow larger and faster than in their native range.	Establishing biological control agents is a time consuming and expensive process. Extremely high numbers of *C. cactorum* egg sticks are required in order for this species to have a significant impact on population densities. Using *Dactylopius* spp. for control is dependent on climate, thus biological control agents are not suitable for cool climates, or areas with regular rainfall.
